# Trimethyltin-Induced Microglial Activation via NADPH Oxidase and MAPKs Pathway in BV-2 Microglial Cells

**DOI:** 10.1155/2015/729509

**Published:** 2015-06-28

**Authors:** Da Jung Kim, Yong Sik Kim

**Affiliations:** Department of Pharmacology, Seoul National University College of Medicine, 103 Daehakno, Jongno-gu, Seoul 110-799, Republic of Korea

## Abstract

Trimethyltin (TMT) is known as a potent neurotoxicant that causes neuronal cell death and neuroinflammation, particularly in the hippocampus. Microglial activation is one of the prominent pathological features of TMT neurotoxicity. Nevertheless, it remains unclear how microglial activation occurs in TMT intoxication. In this study, we aimed to investigate the signaling pathways in TMT-induced microglial activation using BV-2 murine microglial cells. Our results revealed that TMT generates reactive oxygen species (ROS) and increases the expression of CD11b and nuclear factor-*κ*B- (NF-*κ*B-) mediated nitric oxide (NO) and tumor necrosis factor- (TNF-) *α* in BV-2 cells. We also observed that NF-*κ*B activation was controlled by p38 and JNK phosphorylation. Moreover, TMT-induced ROS generation occurred via nicotinamide adenine dinucleotide phosphate (NADPH) oxidase in BV-2 cells. Interestingly, treatment with the NADPH oxidase inhibitor apocynin significantly suppressed p38 and JNK phosphorylation and NF-*κ*B activation and ultimately the production of proinflammatory mediators upon TMT exposure. These findings indicate that NADPH oxidase-dependent ROS generation activated p38 and JNK mitogen-activated protein kinases (MAPKs), which then stimulated NF-*κ*B to release proinflammatory mediators in the TMT-treated BV-2 cells.

## 1. Introduction

Organotin compounds, particularly di- and trialkyl tins, are used as stabilizers in polyvinyl chloride (PVC) and as biocides [1]. Poisoning accidents involving these compounds have been reported [2]. Trimethyltin (TMT) is known to be highly neurotoxic compared to other organotin compounds. Human exposure to TMT causes neuropathological symptoms, cognitive impairments, hyperactivity, aggressive behavior, and seizures [1, 3]. These clinical symptoms are closely related to limbic system dysfunction in the brain [4]. In rodent models, TMT administration induces massive neuronal cell loss with glial reactivity in the brain and behavioral alternations that include cognitive impairments, hyperactivity, and tonic-clonic seizures [4–6]. However, the precise mechanism governing its neurotoxicity remains unclear. Thus far, the suggestions regarding TMT-induced neurotoxic mechanisms include calcium overload [4], excitotoxicity [7], mitochondrial dysfunction [6], oxidative stress [4, 6], and neuroinflammation [5, 8]. Among these neurotoxic mechanisms, neuroinflammation has recently emerged as a key player because extensive glial activation and proinflammatory cytokine production are accompanied by neuronal death in many neurodegenerative disorders [9, 10].

In the central nervous system (CNS), microglia are the resident macrophage-like cells and play a large role in immune system [11]. Lipopolysaccharide (LPS) [12, 13] and various neurotoxins such as amyloid beta (A*β*) [13, 14], 1-methyl-4-phenyl-1,2,3,6-tetrahydropyridine (MPTP) [15], and rotenone [16] can activate microglia to release cytotoxic factors, such as superoxide (O_2_^−^), nitric oxide (NO), tumor necrosis factor- (TNF-) *α*, and interleukin- (IL-) 1*β* [15, 17], factors that reliably trigger neuronal death [15, 18]. The proinflammatory products from activated microglia are generally known to appear via mitogen-activated protein kinases (MAPKs) and the NF-*κ*B pathway [19, 20]. The association between neuroinflammation and microglial activation was elucidated by studies in Alzheimer's disease [9], Parkinson's disease [10], and multiple sclerosis [21].

The administration of TMT to rodents induces early, pronounced glial reactivity with neuronal death, and the enhancement of inflammatory factors including TNF-*α*, IL-1*β*, IL-12, IL-23, and NO in the hippocampus [5, 6]. Previously, it was reported that amoeboid microglia with elevated mRNA levels of proinflammatory factors, such as TNF-*α* and macrophage inflammatory protein- (MIP-) 1*α*, were detected at an early time point after TMT treatment [22]. In the rat hippocampal slice culture [6] and mixed neuronal cultures [23], selective microglial changes prior to any sign of neuronal damage have been reported. Moreover, the microglial activation induced by TMT potentiates neuronal cell death in a coculture of neurons with microglia [24]. Similarly, it has also been reported that TMT can evoke microglial activation in cocultures of microglia with astrocytes [25, 26]. These* in vitro* and* in vivo *studies indicate the importance of cell-to-cell interactions, particularly between microglia and neurons and between microglia and astrocytes in TMT-induced neuroinflammatory reactions [4, 5, 24].

However, few studies have examined the underlying mechanism of direct activation in microglia by TMT. From this background, we aimed to evaluate whether TMT can induce the microglial activation and what kinds of signaling pathways are involved in this process using BV-2 microglial cells.

## 2. Materials and Methods

### 2.1. Reagents

Dulbecco's modified Eagle's medium (DMEM), phosphate-buffered saline (PBS), Hank's balanced salt solution (HBSS), fetal bovine serum (FBS), normal goat serum (NGS), and antibiotic-antimycotic were purchased from Gibco (Grand Island, NY, USA). Trimethyltin chloride, 2,7-dichlorofluorescin diacetate (DCFH-DA), 1-(4,5-dimethylthiazol-2-yl)-3,5-diphenylformazan (MTT), dimethyl sulfoxide (DMSO), bovine serum albumin (BSA), Tween-20, Hoechst 33258, SB203580, SP600125, apocynin, and BAY11-7082 were from Sigma-Aldrich (St. Louis, MO, USA). Rabbit antibodies against p-JNK, p-p38, p-ERK, p-I*κ*B*α*, p38, and I*κ*B*α* were purchased from Cell Signaling Technologies, Inc. (Beverly, MA, USA). Rabbit anti-JNK, ERK, and NF-*κ*B p65 antibodies were from Santa Cruz Biotechnology (Santa, CA, USA). Mouse anti-inducible nitric oxide synthase (iNOS) was from BD Biosciences (Franklin Lakes, NJ, USA). Mouse anti-actin was purchased from EMD Millipore (Billerica, MA, USA). Rat anti-CD11b was from AbD Serotec (Oxford, UK). The goat anti-mouse, -rabbit, and -rat IgG (HRP-conjugated) secondary antibodies were from Enzo Life Science (Farmingdale, NY, USA). All other chemicals were purchased from Sigma-Aldrich.

### 2.2. Cell Culture and Treatment

The murine microglial BV-2 cells (generated from primary microglia transfected with a v-raf/v-myc oncogene [27]) were a gift from Dr. Sang-Kyu Ye (Seoul National University, Seoul, Korea). The cells were maintained at 36°C in a 5% CO_2_ incubator with high-glucose DMEM supplemented with 10% (v/v) heat-inactivated FBS, 1x antibiotic-antimycotic (consisting of 100 units/mL penicillin, 100 *μ*g/mL streptomycin, and 0.25 *μ*g/mL amphotericin B), 2 mM L-glutamine, and 1 mM pyruvate at pH 7.4. At 80% confluence, the cells were harvested for subculture. The cells were seeded on a culture plate and incubated overnight in the culture medium containing 10% (v/v) heat-inactivated FBS and antibiotics. For the western blot, ROS measurement and immunocytochemistry experiments, the media were then replaced with low-glucose DMEM without FBS and antibiotics and incubated for at least 4 hr prior to various inhibitor treatments. For all of the inhibitors, including SB203580 (p38 MAPK inhibitor), SP600125 (JNK MAPK inhibitor), apocynin (nicotinamide adenine dinucleotide phosphate (NADPH) oxidase inhibitor) and BAY11-7082 (I*κ*B*α* phosphorylation inhibitor), and the vehicle (0.1% DMSO), the pretreatments lasted 1 hr, and TMT dissolved in saline was then added to the culture media for the indicated times.

### 2.3. Cell Viability: MTT

To determine the survival of the BV-2 cells upon TMT exposure, 1.5 × 10^4^ cells were seeded in each well of a 96-well tissue culture plate (BD, Franklin Lakes, NJ, USA). After being left overnight, the cells were gently washed with PBS (pH 7.4) twice, and the medium was replaced with low-glucose DMEM medium with 1% (v/v) FBS. Various concentrations of TMT were applied to the BV-2 cells. After 24 hr of incubation, the medium was removed, and the MTT solution (final concentration, 0.5 mg/mL) was added. Following 3 hr of incubation in a CO_2_ incubator at 36°C, the MTT solution was aspirated, and 200 *μ*L of DMSO was added to each well. The absorbances were then read with a microplate reader (Tecan infinite M200 Pro, Tecan, San Francisco, USA) at 495 nm. The absorbance of the control cells was set to 100%.

### 2.4. Measurement of ROS

Intracellular ROS generation was measured with dichlorofluorescein diacetate (DCFH-DA) assays [28]. In 60 mm^2^ tissue culture dishes (BD, Franklin Lakes, NJ, USA), the treated cells at a density of 1 × 10^6^ cells/mL were washed twice with prewarmed HBSS. Next, the cells were incubated with 15 *μ*M DCFH-DA at 36°C in a CO_2_ incubator for 30 min. The cells were again washed twice. A Canto Flow Cytometer (BD Biosciences, CA, USA) was used to fluorescently quantify the cells (excitation at 488 nm and emission at 510 nm). To provide statistical data, the value measured for the control was set to 100%.

### 2.5. Western Blot Analysis

The treated cells were lysed with radioimmunoprecipitation assay (RIPA) buffer (Elpis Biotech, Daejeon, Korea) supplemented with protease and phosphatase inhibitor cocktails (Roche Diagnostics, Rotkreuz, Switzerland) and centrifuged at 14,000 g for 20 min at 4°C. The supernatant was collected, and Bradford assays were used to measure the protein concentrations. Equal protein amounts (20 *μ*g) were separated by 10% (W/V) sodium dodecyl sulfate (SDS) polyacrylamide gel electrophoresis and then transferred onto 0.45 *μ*m pore size nitrocellulose membrane (Bio-Rad Laboratories, Hercules, CA, USA) for 1 hr at 100 V. After 1 hr of blocking in 5% skim milk dissolved in 0.1% Tween-20 containing Tris-buffered saline (TBST) at pH 7.4 for 1 hr at room temperature, the membranes were incubated overnight at 4°C with primary antibodies against p-p38 (1 : 1000), p-JNK (1 : 1000), p-ERK (1 : 2000), p-I*κ*B*α* (1 : 2000), p38 (1 : 2000), JNK (1 : 2000), ERK (1 : 2000), I*κ*B*α* (1 : 2000), iNOS (1 : 1000), and actin (1 : 2000). After washing with TBST three times for 10 min each, the membrane was incubated with goat anti-rabbit IgG-horseradish peroxidase (HRP) or anti-mouse IgG-HRP for 1 hr and then rinsed three times with TBST. The blot was immunolabeled with enhanced chemiluminescence HRP substrate (Thermo Fisher Scientific Inc., Rockford, IL, USA), and a ChemiDoc XRS plus (Bio-Rad Laboratories, Hercules, CA, USA) was used to analyze the immunoblot. Actin was used as the loading control for the total protein.

### 2.6. Immunocytochemistry

To confirm the NF-*κ*B activation induced by TMT, the translocation of the NF-*κ*B p65 subunit was observed via an immunocytochemistry method. Additionally, CD11b immunofluorescence was detected to examine the differences in expression between the different groups. CD11b is a cell surface molecule of microglia that is increased in the activated microglia and has been widely used as a marker in microglial activation [12, 18]. Briefly, 1.5 × 10^4^ cells were seeded on poly-L-lysine-coated glass coverslips. After 12 hr of TMT treatment, fixation with 4% paraformaldehyde was performed at room temperature for 15 min. The coverslips were washed three times with PBS and blocked with PBS containing 3% BSA, 0.3% Triton X-100, and 10% NGS for 1 hr. Next, the cells were stained overnight with the following primary antibodies: rabbit polyclonal NF-*κ*B p65 (1 : 100) (Santa Cruz) or rat polyclonal CD11b (1 : 100) (AbD Serotec). Following three washes with PBS, the coverslips were incubated with fluorescein isothiocyanate- (FITC-) conjugated goat anti-rabbit or donkey anti-rat IgG antibody (1 : 200) for 1 hr. Hoechst 33258 was added to the slides 15 min prior to finishing the incubation with the secondary antibody. After three additional rinses, the coverslips were placed on the glass slides with an antifading mounting medium (Invitrogen, Carlsbad, CA, USA). Immunofluorescence images were obtained from a fluorescence microscope (Axioskop 40; Carl Zeiss, Jena, Germany) at 400x magnification.

### 2.7. Measurement of NO and TNF-*α* Release in the Culture Medium

In a 24-well tissue culture plate (Thermo Fisher Scientific Inc.), 2.5 × 10^4^ cells were treated with TMT in low-glucose DMEM with 1% (v/v) FBS for 24 hr. Then, the medium was transferred and centrifuged at 500 g for 5 min at 4°C. The supernatant fraction was collected for use in the measurements of NO and TNF-*α*. For the NO measurements, a general protocol that has been described previously was followed [29]. Briefly, 90 *μ*L of each sample and 10 *μ*L of Griess reagent (containing 0.1% N-[1-naphthyl] ethylenediamine dihydrochloride in 5% H_3_PO_4_ with 1% of sulfanilic acid) were placed in 96-well tissue culture plate (BD Biosciences). For the standard values, different concentrations of sodium nitrite solution and Griess reagent were placed into the plate. The plate was then gently shaken for 30 min. Absorbance was read in a microplate reader at 540 nm. The nitrite concentration of each sample was calculated from the standard curve. To measure the amount of released TNF-*α*, a TNF-*α* enzyme-linked immunosorbent assay (ELISA) kit was obtained from Abcam (Cambridge, UK). Each dilution of the standard and each sample were placed in the plate, and subsequent experiments were performed according to the manufacturer's protocol. The TNF-*α* measurements were collected using a microplate reader at 450 nm. The concentration of each sample was calculated from the standard curve.

### 2.8. Statistical Analyses

The data are presented as mean ± SEM. GraphPad Prism version 5.0 (San Diego, CA, USA) for Windows was used to analyze the data. The one-way analyses of variance (ANOVAs) with Tukey's multiple comparison test were used to examine the differences between groups. A *P* value below 0.05 was considered to be statistically significant.

## 3. Results

### 3.1. TMT Stimulated Intracellular ROS Generation in BV-2 Cells

**Figure 1:**
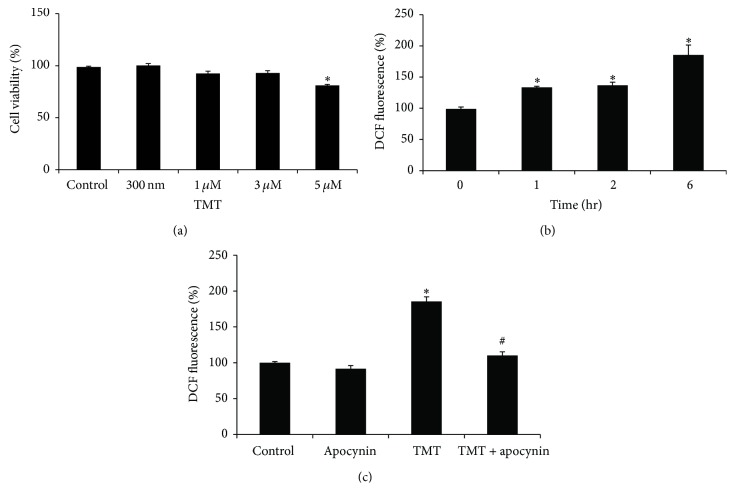
TMT-stimulated ROS generation in BV-2 cells. (a) For the cell viability test, the cells were treated with various concentrations of TMT (300 nM–5 *μ*M) or vehicle (saline) for 24 hr; then MTT assay was performed to measure the cell viability. The value of each sample was normalized to control group. (b) For ROS measurement, 3 *μ*M TMT treated cells was incubated for the indicated periods of time (0–6 hr) and then stained with 15 *μ*M DCFH-DA for 30 min. DCF-fluorescence of each sample was analyzed by the flow cytometry. The value measured at 0 hr was set as 100%. The data are represented as mean ± SEM (*n* = 4). ^*∗*^ *P* < 0.05 compared with control (0 hr). (c) Cells were pretreated with 250 *μ*M apocynin prior to TMT treatment and then incubated for 6 hr. The data are represented as mean ± SEM (*n* = 4). ^*∗*^ *P* < 0.05 compared with the value of control; ^#^ *P* < 0.05 compared with the value of TMT.

BV-2 cells were incubated with various concentrations (300 nM–5 *μ*M) of TMT for 24 hr, and cell viabilities were then evaluated with MTT assays. As shown in Figure 1(a), 300 nM–3 *μ*M TMT did not significantly affect cell viability. The cell viability was 92.9 ± 5.1% at 3 *μ*M TMT, which was decreased to 81.1 ± 2.7% at 5 *μ*M TMT compared with the control group. For further investigations, 3 *μ*M TMT was chosen to rule out the significant cell death induced by TMT.

We initially determined whether TMT could stimulate ROS production in BV-2 cells. Following the TMT exposure, the DCF fluorescence was quantified by flow cytometry at each indicated point in time. Increases in ROS generation were detected within 1 hr of the TMT treatment (Figure 1(b)). A sustained increase in DCF-fluorescence was observed up to 6 hr and reached 1.85-fold that of the control (Figure 1(b)). As NADPH oxidase is known to cause ROS production in macrophage [30, 31] and microglia [15, 28], we next examined whether NADPH oxidase might be involved in TMT-induced ROS generation in BV-2 cells. To inhibit the enzyme activity, 250 *μ*M of apocynin was applied 1 hr prior to TMT treatment. Consequently, the intracellular ROS induced by TMT at 6 hr was markedly diminished (Figure 1(c)). These data suggest that NADPH oxidase might play a crucial role in the production of oxidative stress in TMT-treated BV-2 cells.

### 3.2. TMT Activated p38 and JNK MAPK in BV-2 Cells

**Figure 2:**
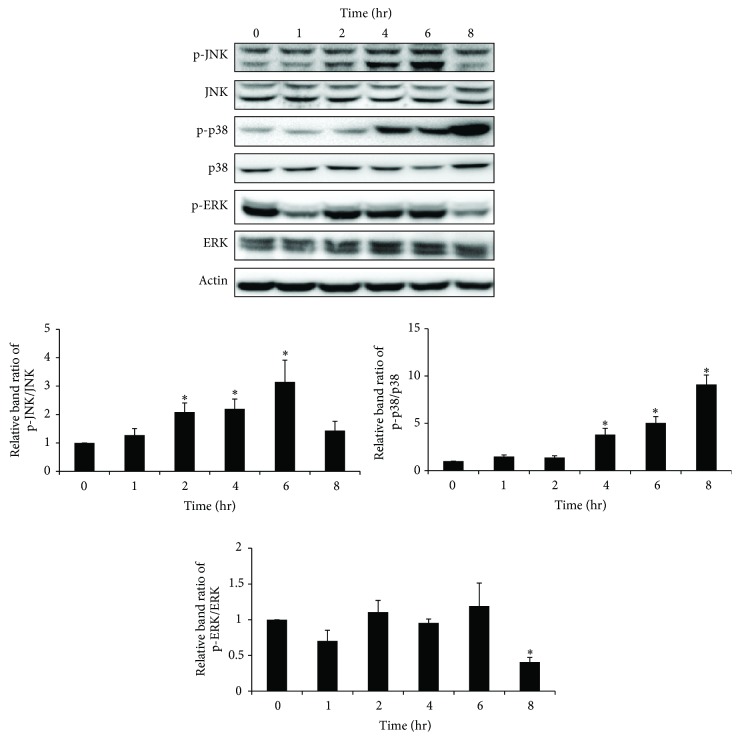
p38 and JNK were activated by TMT exposure in BV-2 cells. Treated cells were analyzed by western blot at each time period. The bar graphs represent the band intensity of each phosphoform of MAPKs normalized to total. The data are represented as mean ± SEM (*n* = 5). ^*∗*^ *P* < 0.05 compared with the control group.

We examined whether TMT could activate MAPK signaling cascades in BV-2 cells. The treated cells were then subjected to western blot analyses at each of the indicated time points. As shown in Figure 2, TMT led to significant increases in p-JNK at 2, 4, and 6 hr after TMT treatment. p-p38 also gradually increased from 4 hr to 8 hr of TMT treatment. However, the TMT-induced p-ERK exhibited transient changes throughout the time course and decreased at 8 hr after TMT treatment.

### 3.3. TMT Exposure Activated NF-*κ*B Signaling in BV-2 Cells

**Figure 3:**
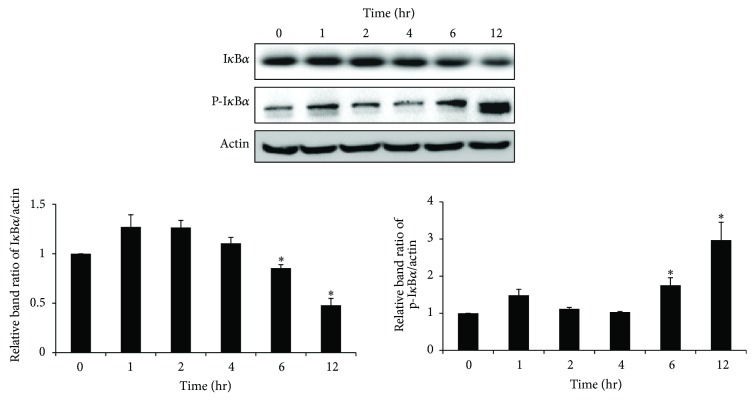
NF-*κ*B signaling pathway was activated by TMT exposure in BV-2 cells. Treated cells were subjected to western blot analysis. The bar graphs represent the band intensity of each protein normalized to actin. The data are represented as mean ± SEM (*n* = 5). ^*∗*^ *P* < 0.05 compared with the control group.

In activated microglia, NF-*κ*B signaling is considered to participate in inflammatory processes that result in the expression of inflammatory mediators, including iNOS and some cytokines, such as TNF-*α* and IL-1*β* [15]. However, it has not yet been reported whether TMT regulates NF-*κ*B activation in BV-2 cells. We further determined the alternations of NF-*κ*B signaling that followed exposure to TMT. Because I*κ*B*α* is known to be an inhibitory subunit of the NF-*κ*B complex that prevents the nuclear translocation of NF-*κ*B, the expression levels of I*κ*B*α* and phospho-I*κ*B*α* protein were analyzed by western blot. Consequently, progressive trends of I*κ*B*α* degradation and p-I*κ*B*α* elevation were detected over time (Figure 3). Typically, at 6 and 12 hr after TMT treatment, I*κ*B*α* phosphorylation and the degradation of I*κ*B*α* were most apparent (Figure 3); these findings indicate that the TMT induced NF-*κ*B activation in BV-2 cells.

### 3.4. p38 and JNK Activations Occur Earlier Than NF-*κ*B Activation in TMT-Treated BV-2 Cells

**Figure 4:**
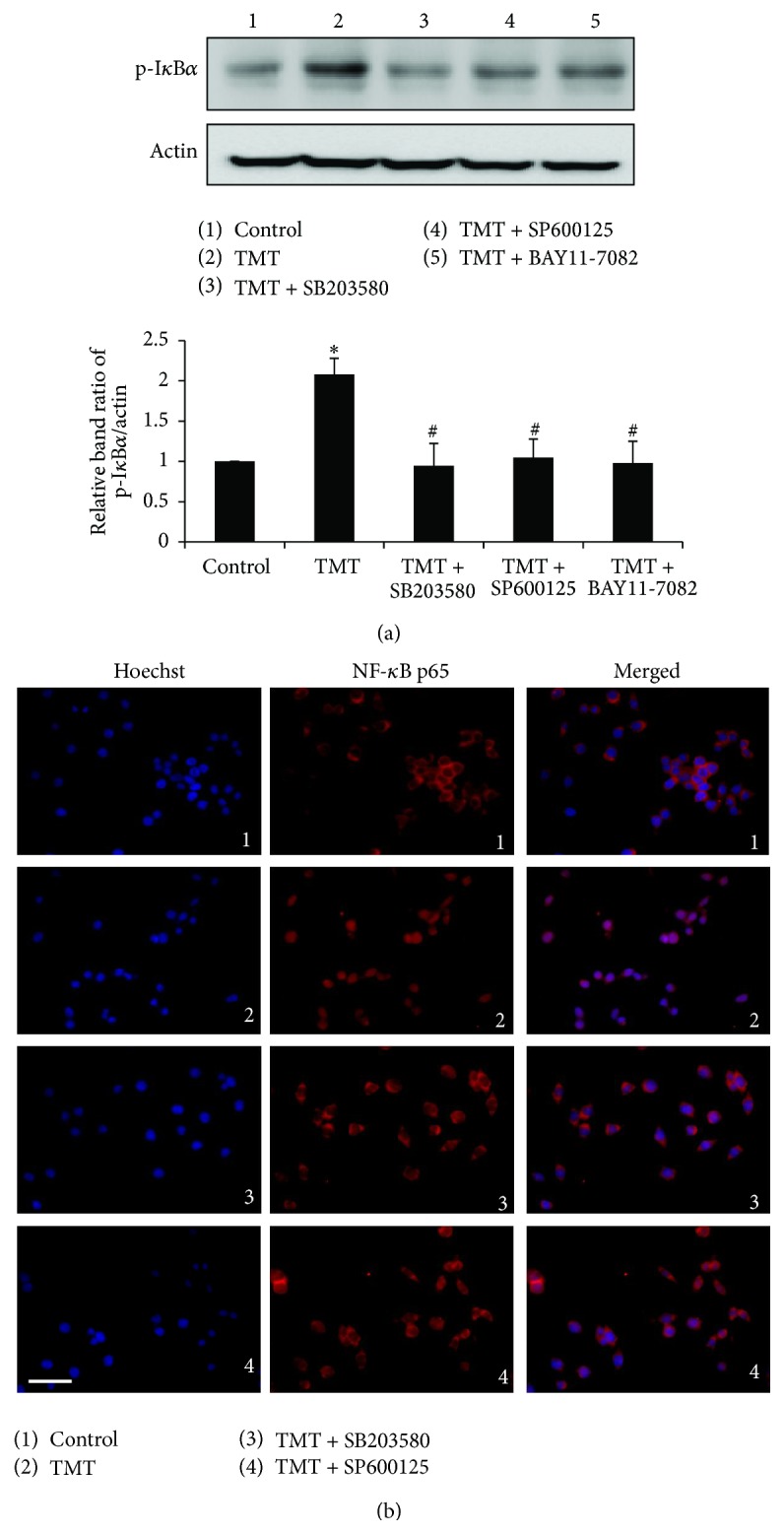
TMT-induced NF-*κ*B activation was reversed by p38 and JNK inhibitor in BV-2 cells. SB203580, SP600125, and BAY11-7082 were pretreated for 1 hr and incubated with TMT for 12 hr. (a) Protein expression of phospho-I*κ*B*α* was shown by western blot. The bar graph represents the band intensity of phosphoform normalized to actin. The data are represented as mean ± SEM (*n* = 5). ^*∗*^ *P* < 0.05 compared with the control group. (b) Following the fixation of treated cells, immunocytochemistry method was carried to observe the translocation of NF-*κ*B p65 subunit morphologically. NF-*κ*B p65 subunit was detected by red fluorescence (Alexa 568), nuclei were stained with blue fluorescence (Hoechst 33258), and the two different types of images were merged. The experiments were repeated more than 3 times. Scale bar: 20 *μ*m.

Next, to examine the involvement of MAPKs on TMT-induced NF-*κ*B activation, SB203580 (p38 MAPK inhibitor), SP600125 (JNK MAPK inhibitor), and BAY11-7082 (I*κ*B*α* phosphorylation inhibitor) were applied prior to the TMT treatment. As shown in Figure 4(a), western blot analyses revealed that TMT significantly elevated p-I*κ*B*α* expression and that its expression was suppressed by treatment with BAY11-7082. In the same experimental conditions, SB203580 and SP600125 also reduced the TMT-induced elevation of p-I*κ*B*α*. Furthermore, the translocation of the NF-*κ*B p65 subunit into the nucleus was determined after 12 hr of TMT treatment by immunofluorescence staining (Figure 4(b)). As shown in Figure 4(b), the NF-*κ*B p65 subunits were localized in the cytosol in the controls (Figure 4(b)-1); however, once the cells were stimulated with TMT, the subunits translocated from the cytosol into the nucleus (Figure 4(b)-2). However, pretreatment with SB203580 (Figure 4(b)-3) or SP600125 (Figure 4(b)-4) inhibited the nuclear translocation of the NF-*κ*B p65 subunits that was induced by TMT treatment. These data suggest that the activation of NF-*κ*B was regulated by MAPKs, particularly p38 and JNK, in TMT-treated BV-2 cells.

**Figure 5:**
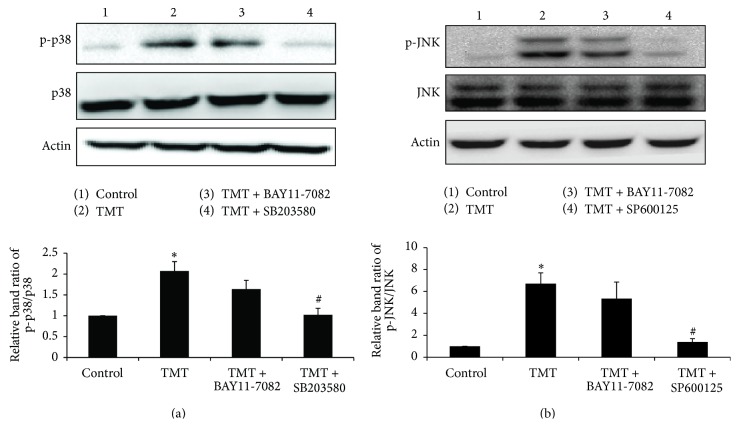
p38 and JNK phosphorylation were followed by NF-*κ*B activation upon the TMT exposure in BV-2 cells. Treated cells were collected at 6 hr and were subjected to western blot analysis. The bar graphs represent the band intensity of each phosphoform normalized to total. The data are represented as mean ± SEM (*n* = 5). ^*∗*^ *P* < 0.05 compared with the control group; ^#^ *P* < 0.05 compared with the value of TMT.

According to the data shown in Figure 4, upon TMT exposure, NF-*κ*B activation by p38 and JNK was confirmed by comparing the alterations in p-I*κ*B*α* and NF-*κ*B p65 nuclear translocation. The regulation of TMT-induced MAPK activation was then examined using BAY11-7082. Consequently, the levels of p-p38 and p-JNK were not affected by BAY11-7082, but each MAPK inhibitor (i.e., SB203580 and SP600125) markedly blocked its expression at 6 hr after TMT treatment (Figures 5(a) and 5(b)). These data suggest that MAPK activation is upstream of the NF-*κ*B pathway. Together, these findings suggest that the TMT-induced p38 and JNK activations occurred prior to NF-*κ*B activation in BV-2 cells.

### 3.5. TMT Induced Increases in iNOS Expression and the Production of NO and TNF-*α* in BV-2 Cells

**Figure 6:**
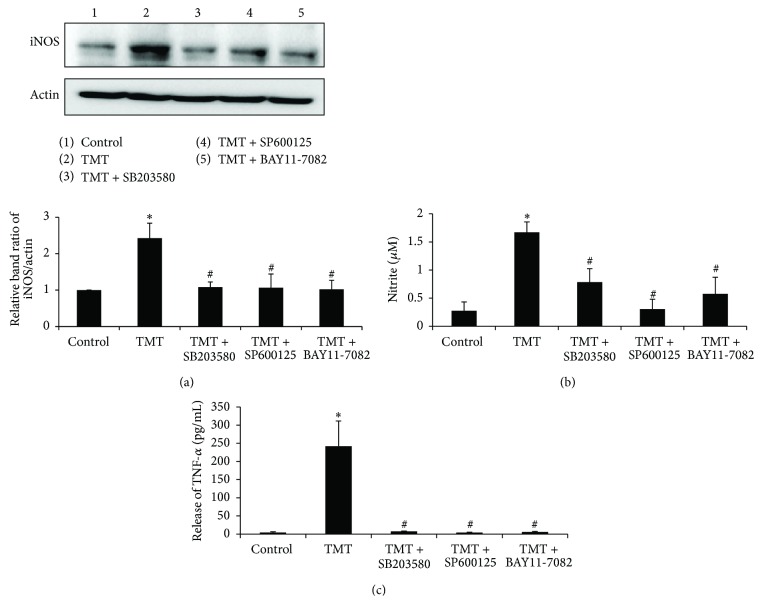
TMT increased iNOS expression and the production of NO and TNF-*α* in BV-2 cells. (a) At 12 hr, the expression of iNOS was analyzed by western blot. The bar graphs represent the band intensity of each protein normalized to actin. (b) At 24 hr, the culture media were taken to assess the level of NO by Griess method. (c) The culture media were also transferred into TNF-*α* Elisa kit to measure the level of TNF-*α* released from the treated cells. The concentration of TNF-*α* (pg/mL) was measured by standard curve. The data are represented as mean ± SEM (*n* = 5–7). ^*∗*^ *P* < 0.05 compared with the control group; ^#^ *P* < 0.05 compared with the value of TMT.

The levels of some proinflammatory molecules, such as NO and TNF-*α*, were then examined in the culture media after the TMT treatments of BV-2 cells because these factors have been reported to participate with NF-*κ*B activation in the responses to various stimuli [15, 17]. First, we examined iNOS expression at 12 hr by western blot analysis (Figure 6(a)). TMT significantly increased iNOS expression by approximately 2.5-fold compared to the control; however, this effect was reversed by pretreatment with SB203580, SP600125, or BAY11-7082. Next, the amounts of released NO at 24 hr were quantified using the Griess reagent method (Figure 6(b)). TMT treatment elevated NO production by approximately 6-fold compared to that observed in the control (control; 0.28 ± 0.16 *μ*M, TMT; 1.67 ± 0.18 *μ*M). Pretreatment with SB203580 reduced the TMT-stimulated NO production by approximately 53%, from 1.67 ± 0.18 *μ*M to 0.79 ± 0.24 *μ*M. SP600125 and BAY11-7082 have also attenuated the TMT-induced NO level to 0.31 ± 0.17 *μ*M and 0.58 ± 0.29 *μ*M, respectively. Moreover, TNF-*α* secretion from the treated BV-2 cells was determined at 24 hr in an ELISA. Continuous TNF-*α* secretion resulted from TMT-treatment and reached 53-fold the control level. However, the massive increase in TNF-*α* from TMT treatment was significantly reversed by pretreatment with SB203580, SP600125, or BAY11-7082 (Figure 6(c)).

### 3.6. CD11b Expression Was Increased in the TMT-Treated BV-2 Cells

**Figure 7:**
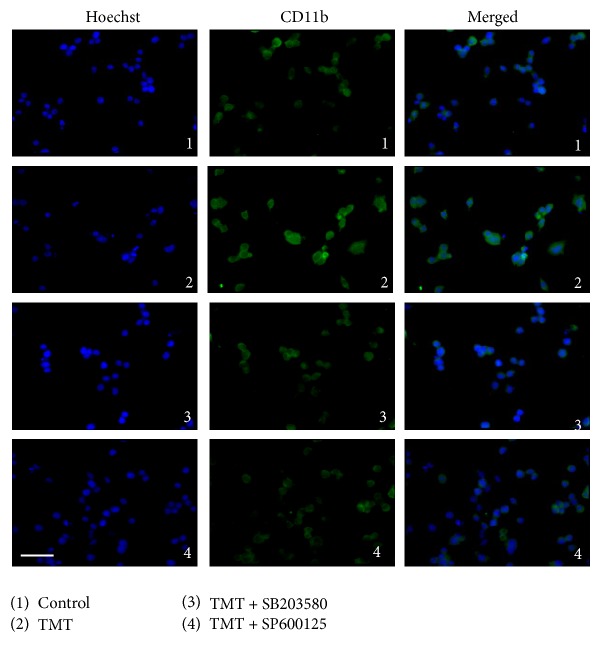
TMT enhanced the expression of CD11b in BV-2 cells. The cells were treated and, after 12 hr, immunocytochemistry was carried to observe the change of CD11b expression among different groups. CD11b was detected by green fluorescence (Alexa 488) and nuclei were stained with blue fluorescence (Hoechst 33258). The two different types of images were merged. The experiments were repeated more than 3 times. Scale bar: 20 *μ*m.

CD11b and other microglial markers, including CD11a, CD11c, CD18, and others, have been reported to prominently appear in many neurodegenerative diseases [32]. Hence, we visualized the activated microglial cells with CD11b immunostaining at 12 hr after TMT treatment. As shown in Figure 7-1, a faint cytoplasmic staining for CD11b can be observed in the control. Following TMT treatment (Figure 7-2), green fluorescence was detected more intensively compared to Figures 7-1, 7-3, and 7-4 and SB203580 and SP600125 prevented the elevation of CD11b expression that resulted from TMT treatment. These results support the notion that TMT elicited microglial activation by increasing CD11b surface molecules in BV-2 cells and that the p38 and JNK MAPK activations induced by TMT contributed to this increase in CD11b expression.

### 3.7. The NADPH Oxidase Inhibitor Apocynin Prevented the TMT-Induced Activations of MAPKs and NF-*κ*B in the BV-2 Cells

**Figure 8:**
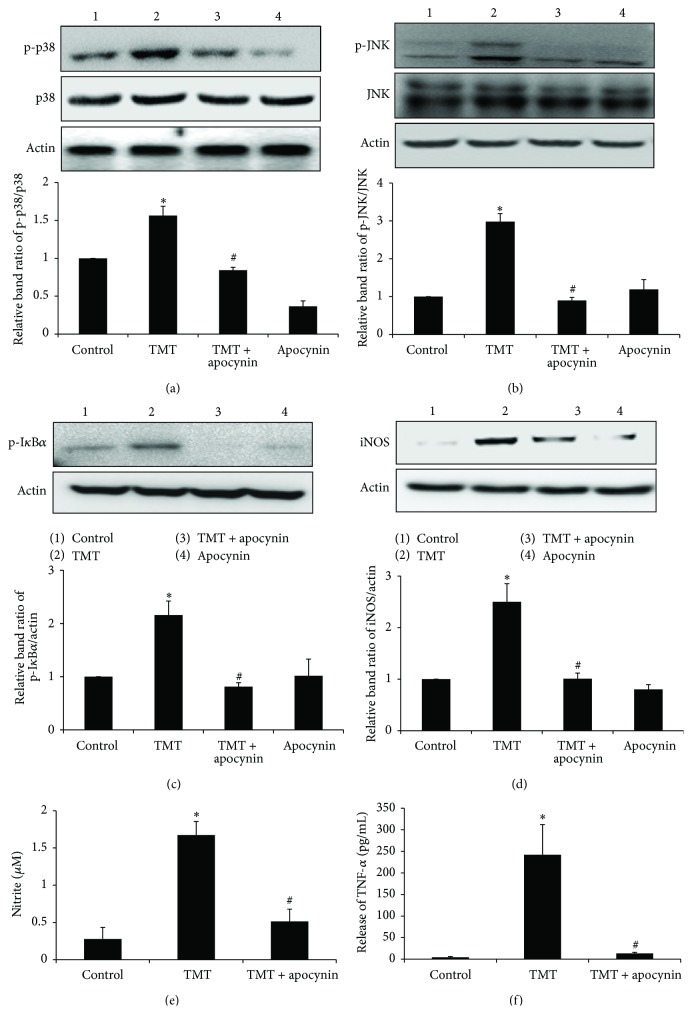
Apocynin suppressed TMT-induced MAPKs activations, p-IKB, iNOS, NO production, and TNF-*α* release in BV-2 cells. (a)–(d) The treated cells were subjected to western blot analysis to assess the expression of phosphorylated p38 and JNK at 6 hr and p-I*κ*B*α* and iNOS at 12 hr. The bar graphs represent the band intensity of each protein form normalized to total. For (e) and (f), the culture media of treated cells at 24 hr were collected and used for (e) NO production measurement by Griess reagent method and (f) TNF-*α* detection by using Elisa kit. The data are represented as mean ± SEM (*n* = 4–7) ^*∗*^ *P* < 0.05 compared with the control group; ^#^ *P* < 0.05 compared with the value of TMT.

Our observations revealed that NADPH oxidase-dependent ROS generation occurred in BV-2 cells and that treatment with apocynin remarkably suppressed the TMT-induced oxidative stress. Therefore, we further investigated the role of toxin-induced ROS generation in microglial activation. Importantly, reductions in NADPH oxidase activity mediated by apocynin treatment decreased TMT-induced p-p38 and p-JNK at 6 hr after TMT treatment (Figures 8(a) and 8(b)). Apocynin also inhibited the downstream signal of TMT-induced MAPK activation and NF-*κ*B activation, as shown by the western blot analyses of p-I*κ*B*α* and iNOS at 12 hr (Figures 8(c) and 8(d)), and consequently reduced TMT-induced NO (approximately 70%) and TNF-*α* (approximately 94%) after 24 hr of TMT treatment (Figures 8(e) and 8(f)). Thus, these data suggest that NADPH oxidase activity was the major source of TMT-induced ROS generation and that intracellular ROS signaling is an upstream effector in BV-2 microglial activation.

## 4. Discussion

Microglia are the innate immune cells of the CNS and play a crucial role in host defense against various invaders [15]. In response to various stimuli, microglia can be activated, which can include the following changes: (1) their morphologies can become ramified, amoeboid, or phagocytic [17, 24]; (2) expression of cell surface antigens, including CD11b, Iba-1, and OX-42 [18, 28]; and (3) production of bioactive factors, such as NO, O_2_^−^, prostaglandins (PGs), TNF-*α*, IL-1, IL-12, and IFN-*γ* [15, 17]. In the present study, we initially determined whether TMT could directly activate BV-2 cells. We observed increased production of NO, and TNF-*α* was detected as previously reported by other researchers [5, 6, 24]. Additionally, we found the increased morphological appearance of CD11b in BV-2 cells with TMT treatment. The expression of CD11b, according to previous reports, is increased once microglial cells become activated by various stimuli although it can be detected in resting states [12, 18]. These findings indicated the direct activation of BV-2 cells by TMT exposure. However, some controversial results were previously reported that TMT either does not directly activate [25, 26] or partially activates microglia [8] with or without any significant morphological changes in microglia-enriched culture [24, 33]. These different results have not been precisely explained yet. A few studies have documented that the discrepancies between* in vitro* studies using primary microglia-enriched cultures might have resulted from variations in the resting states of the microglia that depend on the cell isolation and culture maintenance conditions [17, 34, 35]. Furthermore, BV-2 cells are frequently used as a substitute for primary microglia. It has been reported that approximately 90% of the genes of mouse primary microglial and BV-2 cells overlap following LPS treatment and that this cell line is partially activated in resting states [11, 36]. Based on these reasons, it might be beneficial to analyze microglial activation more clearly during TMT toxicity via the use of BV-2 cells rather than other experimental models [25, 26].

To evaluate the underlying signaling pathways involved in the TMT-induced microglial activation, we performed investigations of the NF-*κ*B and MAPKs signaling pathways, which previously have been discussed in numerous studies as upstream effectors that target the production of inflammatory factors in microglia [13, 15]. In the context of TMT, NF-*κ*B activation has been reported in the murine hippocampus [37], human primary astrocytes [33], and a human neuroblastoma cell line [38]. However, TMT-induced NF-*κ*B activation in BV-2 cells has not been demonstrated yet. In our experiments, TMT-enhanced NF-*κ*B activity was observed and inhibiting the activity led to remarkable decreases in NO and TNF-*α* levels. It was partly comparable to the previous study that observed increased level of cytokines such as TNF-*α*, IL-1*β*, and IL-6 [39]. Recently, it was also reported that dibutyltin, another organotin compound that causes severe immunotoxicity and developmental toxicity in animals [40, 41], increased iNOS, TNF-*α*, and IL-6 mRNA levels in BV-2 cells [42].

LPS [12] and many neurotoxins such as rotenone [16], A*β* [14, 43] have been reported to stimulate BV-2 cells via MAPK activations that result in the modulation of inflammatory factors. Relying on previously reported information, we examined whether TMT could activate MAPKs in BV-2 cells. Therefore, p38 and JNK MAPK activations were resulted by TMT. Suppression of these MAPK activity reduced TMT-induced NF-*κ*B activation. In contrast, inhibition of NF-*κ*B activity did not affect on TMT-induced MAPK activations. Next, to elucidate the role of TMT-induced p38 and JNK activations on the production of inflammatory factors in BV-2 cells, further experiments were performed, and remarkable suppressions of TMT-elevated iNOS, NO, and TNF-*α* levels resulted from pretreatment with SB203580, SP600125, or BAY11-7082. Similarly, decreased CD11b expression was observed following inhibition of MAPK activities. These results suggest that, following TMT exposure, MAPK activity occurs upstream of NF-*κ*B activation in BV-2 cells.

In the present study the ROS generation caused by TMT in BV-2 cells was initially examined because TMT-induced intracellular ROS generation has been frequently proposed to be involved in neurotoxicity [7, 44]. The generation of ROS in microglia has been suggested to initiate various signaling pathways that are related to cytotoxic mechanisms, such as NF-*κ*B, MAPKs, and PI3K/AKT signaling cascades [15, 16]. In our experiment, ROS generation was observed within 1 hr of TMT treatment. Because the main route of ROS generation in microglia is known to be mediated through the activity of NADPH oxidase, which is localized on the surfaces of phagocytic cells and is upregulated in response to various stimuli [15, 28], we inhibited microglial NADPH oxidase with apocynin. Apocynin effectively reversed the elevation in the intracellular ROS induced by TMT. Our results are consistent with those of other reports that have illustrated the involvement of NADPH oxidase in ROS generation in response to multiple stimuli in phagocytic cells [15, 28, 30]. We further determined the relationship of NADPH oxidase-dependent ROS generation with signaling pathway on TMT-induced microglial activation. Interestingly, in addition to the reduction in ROS formation, apocynin prevented the influence of TMT on all parameters, including TMT-increased phosphorylation of p38, JNK and I*κ*B*α*, iNOS expression, and NO and TNF-*α* production. Hence, these findings indicate that TMT-induced MAPKs and NF-*κ*B are targeted by intracellular ROS generation in BV-2 cells. The mechanisms involved in regulation of NADPH oxidase have not been evaluated in this study. However, the involvement of PKC in phagocyte NADPH oxidase activation was reported [45]. There are some reports showing that TMT-increased intracellular calcium results in rat hippocampal neurons [46] and TMT-activated PKC leads to cytotoxicity in PC12 cells [47]. From these studies, it can be suggested that PKC and intracellular calcium might be involved in NADPH oxidase activation in TMT-treated BV-2 cells.

## 5. Conclusions

**Figure 9:**
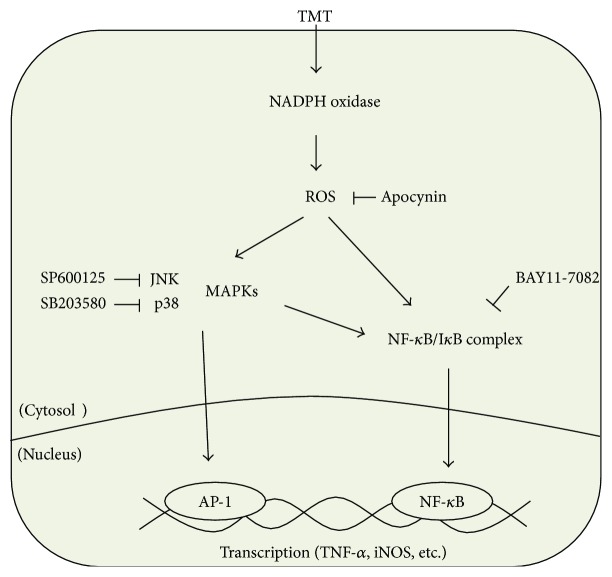
Schematic diagram depicting the possible signaling pathway induced by TMT in BV-2 cells. TMT-induced intracellular ROS may be dominantly generated via NADPH oxidase in BV-2 cells. ROS are then able to activate MAPKs and NF-*κ*B resulting in enhanced production of NO and TNF-*α*. In this experiment, p38 and JNK MAPK activations occurred earlier than NF-*κ*B, and pretreatment with SB203580 or SP600125 suppressed TMT-induced NF-*κ*B activation. Although the mechanism of MAPK regulation in NF-*κ*B activation is not entirely clear, MAPKs may target various protein kinases relating to NF-*κ*B activation and/or transactivation at NF-*κ*B transcriptional level.

Our results showed that TMT-induced oxidative stress mediated p38 and JNK phosphorylation and NF-*κ*B activation resulting in NO and TNF-*α* production in BV-2 cells. But, all of these TMT-induced effects were reversed by the inhibition of NADPH oxidase activity. These results indicate that TMT could directly activate microglial cell via NADPH oxidase-dependent ROS generation. To our knowledge, this is the first report that reveals the direct impact of TMT on BV-2 microglial cells related to underlying mechanisms sequentially (Figure 9). Taken together, it can be suggested that generated ROS and proinflammatory factors from microglia might be involved in TMT-induced neuronal cell death.
